# Laboratory Biomarkers, Cerebral Blood Flow Velocity, and Intellectual Function in Children with Sickle Cell Disease

**DOI:** 10.1155/2020/8181425

**Published:** 2020-02-26

**Authors:** Nataly Apollonsky, Norma B. Lerner, Fengqing Zhang, Deepti Raybagkar, Jennifer Eng, Reem Tarazi

**Affiliations:** ^1^Section of Hematology, St. Christopher's Hospital for Children, Philadelphia, PA, USA; ^2^Division of Blood Diseases and Resources, National Heart, Lung, and Blood Institute/NIH, Bethesda, Maryland, USA; ^3^Department of Psychology, Drexel University, Philadelphia, PA, USA; ^4^Department of Psychiatry, Drexel University College of Medicine, Philadelphia, PA, USA

## Abstract

**Objective:**

The aim of this preliminary study was to describe putative markers of cerebral vasculopathy and investigate relationships among these markers, demographic factors, and cognitive function in a young sample of neurologically normal children with SCD. *Study Design*. Thirty-eight children with homozygous HbS, aged 4–11 years, were included. Estimated IQ and markers of coagulation and endothelial activation, hemolysis, and inflammation, as well as transcranial Doppler velocities, hydroxyurea use, and demographic information were obtained.

**Results:**

Using multiple regression analyses, there were few significant independent associations between biomarkers or blood flow velocity and estimated IQ. Lactic dehydrogenase (LDH) independently predicted cognitive function, but blood flow velocity did not mediate this relationship. Maternal education, patient age, and hydroxyurea status were independent predictors of cognition. Given the small sample size, a LASSO statistical model was employed to further identify potential predictors of IQ, which identified LDH, absolute neutrophil count (ANC), platelet count, thrombin-antithrombin (TAT), tissue factor (TF), maternal education, age, and hydroxyurea as potential predictors of cognition.

**Conclusions:**

In addition to effects of age and maternal education, some vasculopathic markers are associated with cognitive function in young children with SCD, and these relationships do not appear to be mediated through blood flow velocity. Although the lack of association among certain variables was not as predicted, results provide support for further research regarding the influence of vasculopathic markers on cognitive function in children with SCD without stroke, especially intravascular hemolysis and coagulation/endothelial activation, and a possible role for HU treatment in preventing or reversing cognitive decline.

## 1. Introduction

Sickle cell disease (SCD) is one of the most prevalent hematological genetic disorders, which could affect multiple organ systems, including the central nervous system (CNS). Most common CNS manifestations include overt and silent stroke and neurocognitive dysfunction. Overt stroke is caused by large vessel disease. In studied newborn cohorts, the risk of overt stroke is up to 11.5% by 18 years of age [1] and 12.8% by 20 years [2]. Silent cerebral infarctions are associated with small vessel disease and occur in up to 37% of children before the age of 14 [3].

Overt stroke risk can be demonstrated by elevation of blood flow velocity measured by transcranial Doppler ultrasonography (TCD). Since the publication of the Stroke Prevention Trial in Sickle Cell Anemia in 1998 and introduction of TCD screening, prophylactic blood transfusions, and hydroxyurea, the incidence of stroke has decreased. McCavit and colleagues calculated incidence rates of hospitalization for stroke and demonstrated that it decreased by 45% from 0.51 per 100 patient years in 1993–1998 to 0.28 per 100 patient years in 1999–2009 [4].

General cognitive function is negatively affected in children with SCD, with highest to lowest average IQ scores in those with normal magnetic resonance imaging (MRI), silent infarctions, and overt strokes, respectively [5]. Even in the absence of MRI abnormalities, patients with SCD can experience neurocognitive problems and decline in performance over time when compared to matched controls [6]. Abnormal blood velocity on TCD appeared to be associated not only with stroke risk but also with impaired cognition. Bakker and colleagues performed a systematic review of studies assessing TCD velocity and general intellectual functioning in different patient populations [7]. Most of 13 studies investigating children with SCD reported a negative association between blood flow velocities and cognition across development, even in very young children with SCD. Strouse and colleagues [8] reported a high correlation between regional cerebral blood flow and intellectual function and suggested that regional cerebral blood flow may be a more sensitive indicator than blood flow velocity. It is important to recognize that much of these data are based on findings in school-age children, with minimal investigation of the potential effects in younger children with SCD.

In addition to blood flow velocity, some other disease-related factors have been found to be associated with neurocognitive dysfunction in children with SCD. Among factors that have been cited are anemia and hypoxia [9–14], with hematocrit emerging as an independent predictor of IQ when MRI is taken into account [6], elevated steady-state white blood cell count [8], and a platelet count above 500,000 [9]. A potential role for inflammatory processes in cognitive outcomes was proposed by Andreotti and colleagues, who demonstrated significant negative correlations between cytokines (IL-4, IL-5, and IL-8) and executive function in children with SCD [15]. Overall, however, there is a dearth of research examining the association of specific vascular processes and cognition in children with SCD, as well as limited investigation on whether these relationships may be mediated by blood flow velocity.

Considering that the risk of cerebrovascular disease is particularly high in very young children with SCD [1] and that cognitive function tends to decline in this population even in the absence of stroke [16], the purpose of this study is to investigate the potential relevance of markers of vasculopathy on cognitive function in a relatively young cohort of high-risk children with SCD. The study also aims to evaluate if laboratory biomarkers are directly associated with general cognitive ability or if these relationships are mediated by cerebral blood flow velocity, measured by TCD. In addition to the markers evaluated in the previous investigations [8–15], we, based on our understanding of pathophysiology of the disease, investigated additional markers of endothelial injury, coagulation activation, and chronic inflammation.

## 2. Methods

### 2.1. Patient Population

The study was conducted at the Marian Anderson Sickle Cell Center at St. Christopher's Hospital for Children. Patients with HbSS or HbS-beta^0^ thalassemia, aged 4 to 11 years, were eligible for the study. The exclusion criteria included prior history of abnormal TCD or overt stroke, use of medications that would interfere with cognitive testing, blood transfusion within 3 months of study participation, and inability to comply with testing procedures. Of the patients with SCD, eighty-two children met the inclusion criteria. Patients who met the inclusion criteria were recruited during a routine sickle cell maintenance visit, as we only collected study blood and urine samples at the time of a scheduled, routine blood draw completed as part of standard clinical care. Forty-eight patients were approached; forty-one families consented and completed study procedures. Three patients were later determined not to meet the inclusion criteria, and their data were dropped from analysis. Thirty-eight patients were included in the final analysis. Although brain MRI/MRA was not a part of the inclusion or exclusion criteria, of the 12 subjects who had a record of a brain MRI/MRA, none had evidence of ischemia or a silent infarct.

### 2.2. Study Procedures

This prospective study was approved by the institution's Institutional Review Board, and informed consent and assent (for children aged 7 years and older) were obtained from parents and children, respectively. Neuropsychological evaluation, which included a brief screen of intellectual function (IQ) as part of a larger battery, was completed at the time of blood/urine collection or scheduled within 2 weeks of blood/urine collection. Most of the samples were obtained on the day of neuropsychological testing. For three patients, CBC and CMP tests were not performed at the time of the clinic visit during which they participated in the research, and a retrospective chart review was pursued to obtain laboratory results at the closest clinic visit. Hemoglobin F levels for patients who were not on hydroxyurea were obtained by a retrospective chart review within a 12-month period. A parent also completed a general demographic questionnaire at the time of data collection.

### 2.3. Laboratory Measurements

Levels of Hb, WBC, absolute neutrophil count (ANC), platelet count, reticulocyte count, lactic dehydrogenase (LDH), aspartate aminotransferase (AST), bilirubin, and fetal hemoglobin (HbF) were obtained as part of routine care. Commercially available enzyme-linked immunosorbent assay kits were used to measure tissue factor, thrombin-antithrombin complexes, high-sensitivity C-reactive protein (hs-CRP), and vascular cell adhesion molecule-1 (VCAM -1), urinary 11-dehydro-thromboxane B2 (11-dTXB2), and erythropoietin (Epo) levels. These samples were analyzed at Children's Hospital & Research Center in Oakland, California.

### 2.4. Transcranial Doppler

TCD screening studies are routinely performed on all patients with HbSS or HbS-beta^0^ thalassemia from age 2 to 18 years. The studies are executed at St. Christopher's Hospital for Children according to the established Stroke Prevention Trial in Sickle Cell Anemia standards. TCD results obtained within 6 months of study participation were included in the analysis, since these studies are completed on a scheduled basis as part of standard clinical care and were not specifically part of the study protocol. All studies were performed in stable condition. The maximum time-averaged maximum mean (TAMM) velocity of the middle cerebral artery (MCA) was primarily evaluated, but in cases where internal carotid artery (ICA) measurements were higher, it was used instead of MCA velocity for primary analyses. Although previous research has focused on MCA values, we opted to include ICA values in cases where ICA velocity was higher than MCA velocity, considering that MCA is the largest branch of the ICA.

### 2.5. Cognitive Screen

Given the age range of our sample, the *Stanford-Binet Intelligence Scales, 5*^*th*^*edition (SB-V)* [13] Abbreviated Battery IQ (ABIQ) was administered by a licensed, clinical psychologist or a trained, supervised, graduate student to provide an estimate of intellectual function (IQ) for participants. This very abbreviated battery consists of the Nonverbal Fluid Reasoning and Verbal Knowledge subtests of the SB-V, and will be subsequently referred to as “estimated IQ.” The SB-V is an assessment of general intellectual ability appropriate for ages 2 to 85+ years, allowing for the use of one test measure for the children in our sample. Because the ABIQ is a combination of the Nonverbal Fluid Reasoning (object series/matrices) and the Verbal Knowledge (vocabulary) subtests, it is not an independent measure. The Nonverbal Fluid Reasoning test, subsequently referred to as “estimated nonverbal IQ,” measures fluid intelligence, or the ability to apply logic and reason to a novel situation or problem. The Verbal Knowledge test, subsequently referred to as “estimated verbal IQ,” measures crystallized intelligence, or the ability to use skills, knowledge, and prior experience, which can be accessed from memory. A combination of fluid reasoning and knowledge measure is recognized as an appropriate estimate of global ability, and these 2 specific measures load highly on fluid and crystallized ability factors, respectively [13]. The correlation between the 2-subtest ABIQ and the 10-subtest Full-Scale IQ of the SB-V is 0.87 for children aged 6 years and above and 0.81 for children aged 2 to 5 years, supporting the use of the abbreviated version for a brief screen of general intellectual ability. Age-referenced standard scores were used in the analyses.

### 2.6. Statistical Analysis

Pearson correlation coefficients were calculated to examine the relationship among biomarkers of hemolysis (LDH, AST, reticulocyte count, and bilirubin), anemia (Hgb and HgbF), inflammation (CRP and WBC), coagulation/endothelial activation (TF, TAT, and sVCAM-1), and 11-dTXB2 and erythropoietin levels. We also evaluated the relationship of these biomarkers with time-averaged maximum mean (TAMM) velocity and estimated IQ. ANOVAs were used to compare IQ scores and TCD velocities based on hydroxyurea status.

Biomarkers were then examined as predictors of IQ using a set of multiple regression models. Standardized regression coefficients were reported. In addition, due to the small sample size and concern that the multiple regression analyses were underpowered, we used an advanced regression-based model called LASSO (least absolute shrinkage and selection operator) to identify potential biomarkers for predicting IQ [17, 18]. This approach is well suited to analyze correlated true and noise predictors by shrinking the coefficients of unimportant predictors to exactly zero [19]. The cross-validation mean squared error (CV MSE) was compared between the LASSO and the saturated model with the goal of reducing the prediction error (decreasing MSE). The saturated model refers to a model where all the predictors are included, which can increase noise from predictors that are not relevant. We then conducted mediation analyses to examine whether cerebral blood flow mediates the relationship between biomarkers and IQ. A bias-corrected 95% confidence interval (CI) was calculated using 10,000 bootstrap samples. Mediation is significant if the CI for the indirect effect does not include zero. For missing data, we performed multiple imputation using Markov chain Monte Carlo algorithms, known as chained equations imputation. Analyses were conducted using R version 3.1.1 and SPSS version 23.

## 3. Results

A summary of the sample characteristics is presented in Table 1. Thirty-eight children, aged 4–11 years (M = 7.19, SD = 1.61), participated in this study, with 53% of the sample being male. Average maternal education was 13.25 years (SD = 1.93). The mean estimated IQ score of the sample fell within the lower end of normal, but ranged from the deficient to high average range. The mean performance on the estimated nonverbal IQ (Nonverbal Fluid Reasoning) and the estimated verbal IQ (Verbal Knowledge) tasks was similar, although they were not meaningfully correlated with each other.

Hematologic and TCD data are presented in Table 1. The TAMM velocity represents the right or left MCA velocity in 84% of cases; for the remaining cases, the right or left ICA value was included. Correlation data for the biomarkers, TAMM values, demographic factors, and IQ scores are presented in Tables 2 and 3. With regard to the association among various biomarkers, hemoglobin was significantly positively correlated with HbF and was negatively correlated with markers of hemolysis (reticulocyte count and bilirubin). HbF was significantly negatively correlated with WBC and with markers of hemolysis (LDH, bilirubin, reticulocyte count, and AST). Markers of coagulation and endothelial activation (TAT, sVCAM, and TF) were not significantly related to one another. TAT and sVCAM were moderately correlated with reticulocyte count. Among markers of inflammation, WBC moderately correlated with markers of hemolysis, as mentioned above. Hs-CRP was not significantly correlated with any other biomarker, including WBC. Epo levels and dTXB2 were highly correlated with one another and with Hb. LDH was the only biomarker correlated with highest TAMM velocity. TAMM velocity was not significantly correlated with the estimated IQ scores.

Analyses comparing participants taking HU versus those who were not taking HU showed a significant difference in estimated nonverbal IQ (*F* *=* 9.02, *p*=0.005), and TCD velocity for the left PCA, with lower velocities in those taking hydroxyurea (*F* *=* 5.22, *p*=0.029). No other group differences were statistically significant.

Multiple regression analyses were used to consider *independent* predictors of cognitive function, including markers of vasculopathy, HU status, age, and maternal education. These analyses revealed that maternal education in years (*β* = 0.34, *p*=0.037) and LDH (*β* = −0.35, *p*=0.036) were significant predictors of estimated IQ. Higher levels of maternal education were related to higher IQ, while higher levels of LDH were associated with lower IQ. Hydroxyurea was positively associated with estimated nonverbal IQ (*β* = 0.79, *p*=0.035), and age in months was negatively associated with estimated nonverbal IQ (*β* = −0.31, *p*=0.046). Maternal education in years (*β* = 0.47, *p*=0.004) was a significant positive predictor of estimated verbal IQ.

Given the relatively small sample size and concern that regression analyses were underpowered, we then used an advanced statistical model LASSO to identify *potential* biomarkers that explain most of the variation in IQ. This approach resulted in the identification of additional vasculopathic markers as potential predictors of cognitive function (Table 4). In addition to maternal education and LDH, potential variables selected by the LASSO for predicting estimated IQ include age in months, EPO, TAT, ANC, and platelet count. The CV MSE was reduced from 2.88 obtained by the saturated model to 0.94 by the LASSO. In addition to HU status and age, LDH and ANC were also selected by the LASSO for predicting estimated nonverbal IQ. The CV MSEs achieved by the saturated model and by the LASSO were 3.23 and 0.96, respectively. For estimated verbal IQ, in addition to maternal education, TF, TAT, dTXB2, and platelet count were identified as possible predictors. The CV MSE was reduced from 2.18 by the saturated model to 0.88 by the LASSO.

Mediation analyses were then conducted to examine whether TAMM velocity mediates the relationship between biomarkers and IQ. LDH (*β* = 0.40, *p*=0.016) was the only significant predictor of the highest TCD value. Mediation analysis revealed that both the total effect (*β* = −0.36, BC 95% bootstrap CI (−0.70, −0.03)) and the direct effect (*β* = −0.38, BC 95% bootstrap CI (−0.75, −0.01)) of LDH on estimated IQ were significant, while the indirect effect (*β* = 0.02, BC 95% bootstrap CI [-0.13, 0.18]) of LDH on estimated IQ through TCD was not significant.

## 4. Discussion

Vascular damage in SCD has been broadly attributed to complex interactions involving intra-erythrocytic hemoglobin polymerization, increased cell rigidity and sickling, hemolysis, anemia, adhesion, endothelial injury, platelet and leukocyte activation, coagulopathy, and chronic inflammation [20]. Hemolysis-associated nitric oxide scavenging by cell-free plasma hemoglobin has also been implicated in the process [21], and increased HbF may be protective. It remains uncertain whether vasculopathy plays a role in neurocognitive problems that are unassociated with detectable clinical or radiographic evidence of stroke. This study represents a preliminary investigation of relationships between markers of hemolysis, endothelial injury, coagulation activation, and inflammation and intellectual function in children with SCD with no history of overt stroke. As elevated TCD velocities have been used as surrogate measures of cerebral vasculopathy, the association of TCD values and cognitive function, and potential mediating effects of TCD velocities on the relationship of biomarkers and cognition was also considered.

Although the aim of this study was not to describe intellectual function in youth with SCD, we observed that the mean estimated IQ of our sample, as represented by the Abbreviated IQ score of the SB-V, fell in the lower end of the average range. IQ standard scores have a mean of 100 and a standard deviation of 15. Of interest, scores on the verbal and nonverbal tasks were not meaningfully correlated, and performance on these tasks appeared to be predicted by different factors, highlighting various demographic and pathophysiological risk factors for cognitive dysfunction in youth with SCD. The mean IQ score of our sample is more consistent with that of matched or sibling control groups in studies evaluating intellectual function in SCD, and slightly higher than the weighted mean IQ of 86.4 for neurologically healthy SCD samples in a meta-analysis [16]. Because intellectual and cognitive functions tend to decline with age in youngsters with SCD [12, 16], a finding which is supported by these study results, the functioning of our sample may reflect the relatively young age compared to other published studies, highlighting the importance of identifying and addressing risk factors for cognitive decline in younger children with SCD. That is, stability of intellectual function in this cross-sectional cohort of young children with SCD is not necessarily expected, given biomedical and demographic risk factors [16, 22].In addition, our results may reflect the use of an abbreviated IQ measure, limiting analysis of skills such as working memory and processing speed in the context of IQ assessment.

Calculated Pearson correlation coefficients and multiple regression analyses failed to demonstrate relationships between cognitive function and previously implicated markers such as low hemoglobin [9–13], elevated WBC [8], and platelet count [9] in the group studied. Measures of inflammation, coagulation activation, and endothelial injury (hs-CRP, TF, sVCAM, and TAT) were also not correlated with or predictive of IQ in these analyses. Among the markers studied, results of regression analyses suggested that only LDH was an independent significant predictor of estimated IQ. While LDH was also correlated with TAMM velocity, its association with IQ was independent and not mediated by this variable. These findings are consistent with previous studies showing that hemolysis is independently associated with lower IQ [9, 23].

Using a more sophisticated modeling approach to address low power, ANC, platelet count, TAT, TF, EPO, and dTXB2 were identified as additional potential biomarkers predicting intellectual function. Such results suggest that inflammation, coagulation activation, and endothelial injury may play a role in compromising neurocognition separate from effects on blood flow velocity, and this warrants further investigation.

Hydroxyurea was identified as a predictor of estimated nonverbal IQ across analyses. These findings are of particular interest, as there has been minimal investigation of cognitive outcomes associated with this treatment. Our results are compatible with those of Puffer and colleagues who demonstrated evidence of HU's cognitive effect in a small sample of HU-treated children with SCD compared with non-treated SCD controls [24]. It is conceivable that the HU effect was mediated through some of its known activities, such as increasing HbF, raising baseline Hb, and lowering WBC, LDH, and red cell adhesion [25]. In our cohort, however, there were very limited correlations between HU status and blood flow velocity (left PCA only), and no significant correlation between HbF or Hb levels and IQ. Nonverbal IQ represents “fluid reasoning,” which is more vulnerable to changes in neurologic health than verbal or crystallized intelligence. This is consistent with findings that nonverbal reasoning may be specifically vulnerable to effects of increasing age in children with SCD. The positive association between treatment with HU and fluid reasoning skills suggests potential protection against cognitive decline or improvement in cognitive function in the context of HU treatment. Further evaluation of HU effect on specific aspects of cognition during different developmental stages is warranted.

Across analyses, maternal education in years was found to be an independent predictor of higher estimated IQ and verbal IQ. Consistent with the approach used in pediatric research in general, and given that all but one of the primary caregivers in this study were mothers and that most of these (66%) were single, widowed, or divorced mothers, we did not analyze paternal education for this study. In the cases where paternal education was reported, it was identical to maternal educational attainment in 71% of the cases. In our previous work, demographic factors, particularly maternal education and family income, appeared to play a more significant role in predicting variance in neuropsychological performance than did disease-related factors [26]. The relationship between socioeconomic status and cognitive functioning in children with SCD without stroke has been highlighted by others [9, 26–28]. A recent study also found an association between maternal financial stress and cognitive function in children with SCD [29]. Older subject age also emerged as a significant predictor of lower estimated IQ and nonverbal IQ, supporting previous findings that cognitive risk increases with age in children with SCD [12, 16] and further highlighting the need to identify both biological and psychosocial risk factors early in development in order to prevent decline.

This study was associated with several limitations. The sample size was relatively small, reflecting the limited age range and genotype chosen. We attempted to compensate small sample size by using an advanced statistical model, LASSO, to identify potentially significant biomarkers. Unfortunately, sample size still limited the power to detect potential relationships and mediating effects in this study. For example, several studies have demonstrated that lower Hb level is associated with decreased cognitive function in patients with SCD [6, 9, 10, 27]. We may have lacked the power to detect this relationship in our study, given that our sample included few patients with a hematocrit below 20. Our cohort was also quite young, ranging in age from 4 to 11 years. Although TCD velocities and stroke risks tend to be highest in young children with SCD, the age range may have compromised our ability to fully detect associations between studied markers and cognitive function, as the effects of inflammation, anemia, and coagulation activation are thought to be cumulative over time [5].

Another important limitation relates to the limited inclusion of cognitive data in this study, which did not allow us to explore potentially significant associations between various biomarkers, cerebral blood flow, and other aspects of cognitive function. The inclusion of limited cognitive data in this preliminary investigation was intended to allow for consideration of potential cognitive effects of a very large number of biomarkers given our small sample size, and to inform future studies by narrowing down relevant biomarkers for further exploration. Performance on a general IQ screen, however, does not allow for an in-depth analysis of the underlying cognitive skills that contribute to task performance, such as attention, working memory, or spatial processing. A limited cognitive screen may have also masked the intra-individual variability associated with SCD-related neurological and cognitive changes and limit sensitivity in identifying more specific effects of these biomedical processes. In addition, testing was most often conducted prior to or following a regular clinic visit and blood draw; therefore, performance may have been compromised by anxiety and/or fatigue. Future studies should investigate potential effects on more specific cognitive skills or domains. Finally, we did not include a comparison or control group since the goal of the study was not to describe cognitive function in a SCD sample but to identify potential physiological predictors of cognitive function in a relatively young cohort of youth with SCD. But lack of a control comparison for the cognitive data limits our ability to understand the degree to which cognitive function in this sample could be accounted for by other demographic factors.

The lack of comprehensive MRI constituted another important study limitation. While children were excluded if they had experienced an overt stroke or an abnormal TCD, neuroimaging data were available on only 12 participants (31%). As a result, associations among specific biomarkers, cognitive dysfunction, and silent stroke risk could not be reliably assessed.

In conclusion, although we did not find many significant associations between markers of inflammation, endothelial activation, and coagulation activation, this preliminary investigation provides some support for future appropriately powered studies investigating these predictive markers of cognitive function in neurologically normal children with SCD. More specifically, this study suggests that hemolysis and coagulation/endothelial activation are related to cognitive health in youth with SCD, and this does not appear to be accounted for by effects on blood flow velocity. In addition, these results suggest potential benefits of treatment with HU as it relates to cognitive development in this at-risk population. Future studies should further direct or indirect effects of vascular health on cognitive development in SCD and employ MRI/MRA evaluations and expanded neurocognitive testing procedures able to measure attention, executive function, memory, visuomotor capabilities, language, and academic achievement [21, 22, 30]. The possibility that HU treatment may prevent or reverse cognitive decline in this population is of particular import and should be further explored.

## Figures and Tables

**Table 1:** Sample characteristics.

	*N* (%)	Mean (SD)	Range
Gender			
Male	20 (53)		
Female	18 (47)		
Age (years)		7.19 (1.61)	4–11
Maternal education (years)		13.25 (1.93)	11–18
SB-V			
ABIQ (standard score)		91.79 (11.97)	70–115
Nonverbal (scaled score)		8.63 (2.57)	3–13
Verbal (scaled score)		8.63 (2.64)	3–14
Hydroxyurea (yes)	10 (26)		
MRI (yes)	12 (32)		
TCD (cm/sec)			
Highest value		131 (22)	92–178
Right MCA		123 (25)	82–178
Left MCA		125 (22)	68–175
Right ACA		54 (12)	30–91
Left ACA		55 (14)	33–88
Right DICA		95 (16)	72–153
Left DICA		96 (20)	71–154
Right PCA		62 (13)	42–94
Left PCA		64 (14)	39–96
Hgb (g/dL)		8.25 (1.00)	6.30–11.00
HbF (%)		11.88 (6.81)	1.40–28.90
WBC (103/*μ*L)		11.06 (3.02)	6.50–19.10
CRP (ng/ml)		1417 (1423)	236–5866
ANC (103/*μ*L)		5.15 (3.04)	1.10–14.80
LDH (U/L)		584 (164)	279–972
Reticulocyte (%)		10.10 (4.72)	2.80–23.00
Bilirubin (mg/dL)		2.87 (1.89)	1.00–10.80
AST (U/L)		46.64 (12.58)	25.00–72.00
EPO		100 (91)	27–485
TF (pg/ml)		248 (111)	78–549
TAT (ng/ml)		78.60 (133.72)	0.10–500.00
sVCAM-1 (ng/ml)		1566 (596)	838–4214
11-dTXB2 (pg/ml)		4556 (2687)	285–10000
Platelet count (103/*μ*L)		428 (114)	152–705

Note: ^*∗*^*p* < 0.05; ^*∗∗*^*p* < 0.01.

**Table 2:** Correlations between biomarkers, TCD, and IQ.

	HbF	WBC	CRP	ANC	TF	EPO	sVCAM	TAT	dTXB2	Platelet	Reticulocyte	LDH	Bilirubin	AST	TCD	ABIQ	NVIQ	VIQ
Hgb	0.77^*∗∗*^	−0.26	−0.12	−0.28	−0.15	−0.53^*∗∗*^	−0.28	0.05	−0.40^*∗*^	0.35^*∗*^	−0.40^*∗*^	−0.38^*∗*^	−0.24	−0.34^*∗*^	−0.29	0.17	0.13	0 .13
HbF		−0.42^*∗∗*^	−0.14	−0.35^*∗*^	−0.24	−0.46^*∗∗*^	−0.26	−0.04	−0.14	0.15	−0.40^*∗*^	−0.50^*∗∗*^	−0.36^*∗*^	−0.41^*∗*^	−0.28	0.08	0.12	0.00
WBC			0.18	0.82^*∗∗*^	0.03	−0.07	0.24	0.12	0.10	−0.11	0.62^*∗∗*^	0.23	0.37^*∗*^	−0.01	0.19	−0.11	−0.00	−0.16
CRP				0.07	−0.14	0.21	0.07	0.23	0.07	−0.03	0.20	−0.03	−0.23	−0.12	0.13	−0.12	−0.06	−0.12
ANC					0.02	0.02	0.45^*∗∗*^	−0.02	0.06	−0.10	0.55^*∗∗*^	0.17	0.34^*∗*^	−0.00	0.16	−0.17	−0.17	−0.10
TF						0.20	−0.28	−0.02	−0.08	0.01	−0.08	0.29	0.07	0.18	0.04	−0.01	−0.22	0.20
EPO							0.21	−0.05	0.34^*∗*^	−0.12	0.11	0.14	0.02	0.37^*∗*^	0.23	−0.18	−0.17	−0.11
sVCAM								.09	0.15	−0.25	0.36^*∗*^	0.05	0.25	0.20	0.28	0.02	−0.02	0.05
TAT									0.04	0.00	0.33^*∗*^	−0.23	−0.17	−0.20	0.03	0.24	0.16	0.21
dTXB2										−0.25	0.14	−0.15	0.00	0.17	0.19	−0.08	0.14	−0.25
Platelet											−0.07	0.03	0.02	−0.03	−0.15	−0.15	−0.13	−0.10
Reticulocyte												−0.11	0.41^*∗*^	0.04	0.00	−0.10	−0.06	−0.10
LDH													0.18	0.51^*∗∗*^	0.40^*∗*^	−0.29	−0.36^*∗*^	−0.09
Bilirubin														0.47^*∗∗*^	0.03	−0.07	−0.12	0.01
AST															0.22	−0.02	−0.11	0.08
TCD																−0.18	−0.04	−0.23
ABIQ																	0.76^*∗∗*^	0.77^*∗∗*^
NVIQ																		0.18

**Table 3:** Correlations between IQ and demographics.

	NVIQ	VIQ	Age	MatEd
ABIQ	0.76^*∗∗*^	0.77^*∗∗*^	−0.15	0.43^*∗*^
NVIQ		0.18	0.37^*∗*^	0.16
VIQ			0.14	0.49^*∗∗*^
Age				0.30

Note*:*^*∗*^*p* < 0.05; ^*∗∗*^*p* < 0.01.

**Table 4:** Selected potential biomarkers by the LASSO model for predicting IQ.

ABIQ	Estimated NVIQ	Estimated VIQ	Standardized coefficient	Predictor	Standardized coefficient
Predictor	Standardized coefficient	Predictor			
Age	−0.043	Age	−0.153	Maternal education	0.233
Maternal education	0.178	ANC	−0.029	TF	0.011
EPO	−0.004	LDH	−0.039	TAT	0.025
TAT	0.036	HU	0.5	dTXB2	−0.06
ANC	−0.026			Platelet	−0.021
Platelet	−0.048				
LDH	−0.064				

## Data Availability

The laboratory data used to support the findings of this study are included within the tables information file.

## Abbreviations

IQ: Intelligence quotient
TCD: Transcranial Doppler ultrasound
LDH: Lactic dehydrogenase
ANC: Absolute neutrophil count
TAT: Thrombin-antithrombin
TF: Tissue factor
SCD: Sickle cell disease
RBC: Red blood cells
MRI: Magnetic resonance imaging
AST: Aspartate aminotransferase
HbF: Fetal hemoglobin
hs-CRP: High-sensitivity C-reactive protein
VCAM -1: Vascular cell adhesion molecule-1
11-dTXB2: 11-dehydro-thromboxane B2
Epo: Erythropoietin levels
ICA: Internal carotid artery
MCA: Middle cerebral artery
ABIQ: Abbreviated battery IQ
NVIQ: Nonverbal IQ
VIQ: Verbal IQ
LASSO: Least absolute shrinkage and selection operator
CV MSE: Cross-validation mean squared error.
